# En_Línea. An online treatment to change lifestyle in overweight and obesity: study protocol for a randomized controlled trial

**DOI:** 10.1186/s12889-019-7928-1

**Published:** 2019-11-21

**Authors:** Carmen Varela, Carmina Saldaña

**Affiliations:** 1 Department of Clinical Psychology and Psychobiology, Faculty of Psychology, University of Barcelona, Passeig Vall d’Hebrón, 171 P.C, 08035 Barcelona, Spain; 2 Institut de Neurociències, University of Barcelona, Passeig de la Vall d’Hebron, 171 P.C, 08035 Barcelona, Spain

**Keywords:** Overweight, Obesity, Randomized controlled trial, Online therapy, Internet, Weight control program

## Abstract

**Background:**

Obesity has become a major public health problem. Innovative treatments are necessary. Internet and new technologies have been reported effective results in weight control programs, especially those with personalized feedback. This paper presents the protocol for a randomized controlled trial to test the effectiveness of an online weight control program, called *en_línea,* comparing with a standard group therapy and a control group.

**Methods:**

This is a randomized controlled trial with three intervention arms: *en_línea,* standard group therapy and control group. To perform this study, 305 adults (18–65 years) with overweight type II (27–29.9 kg/m^2^) or obesity type I (30–34.9 kg/m^2^) will be invited to participate. Interventions will last 17 weeks with follow-ups 1, 3, 6 and 12 months after the post-treatment appointment. The primary outcome will be post-treatment weight loss and the maintenance during the follow-ups. Secondary outcomes will be adherence rates, drop outs and quality of life. Participants will be assessed before randomization and they will be sign an inform consent.

**Discussion:**

The future challenge is to design innovative obesity treatments. Internet could be a useful tool to improve traditional weight control programs. This new intervention format is appropriate for patients who prefer not to share their intimate problems with a group, and for the new generations who feel comfortable using new technologies. Besides, Internet allows reaching a large amount of people at the same time, even if they live far away.

**Trial registration:**

ClinicalTrials.gov NCT04127201. Retrospectively registered 15th October 2019.

## Background

Obesity has become a Western countries public health problem [1]. There was a transition in the last decades and, currently, there are more obese than underweight people over the world [1]. In 2016, according to World Health Organization (WHO), more than 1.9 billion adults were overweight and 650 million of these were obese [2]. People who suffers obesity are more likely to present comorbid diseases like diabetes, cancer or hypertension [3]. Moreover, previous studies have shown an association between obesity and psychological problems like depression, anxiety or stress [4]. Weight discrimination and bias use to be related with psychological problems aforementioned [5, 6].

Obese people need innovative treatment options, previous investigations have developed weight loss programs focused on diet, physical activity and self-recording [7]. These interventions have shown effective short-term weight loss results, but poor adherence rates and long-term results [7–9]. To design effective treatments, it is important to change the focus from treating the problem to treat the person who is suffering the problem, to understand these people and their environment [10].

Traditionally, there was a tendency to blame obese people when they failed or dropped out an intervention [10, 11]. The lack of social support, even by healthcare professionals, is an important barrier to adhere to weight control programs [10, 11]. In general, obese people face an unsupportive environment where the standards of beauty are unrealistic and unreachable, especially for women [12]. The high leisure-time internet use has become another barrier promoting sedentary behaviors [13], and at the same time offering poor quality information about healthy habits to reach the unhealthy thinness ideal [14].

However, internet world usage has grown 1104% since 2000 [15] and, properly-used, could be a potential tool to design innovative interventions [16]. Web-based weight control programs could solve some limitations of traditional interventions. These new tools can reach many people at the same time [17], which is an urgent need [1, 2]. Moreover, the content of these programs must include further areas than nutrition and exercise [7].

Previous reviews have reported effectiveness results of web-based computer-tailored interventions [7–9, 18–20]. These investigations concluded that web-based weight control programs are more effective than control or minimal contact interventions [18, 19]. However, effectiveness results are short-term and adherence is too low comparing with traditional interventions or web-based programs with an extra component, like group reinforcement sessions [7–9, 20]. Web-based interventions with personalized feedback, delivered by a health professional, have reached better results than those without feedback or automatized feedback [9–11]. Therefore, internet could be an innovative and promising way to deliver weight control interventions, but further investigation is need [21].

We have developed a web-based computer-tailored intervention aimed to acquire healthy long-term habits. The body mass index (BMI) of the target population must be between 27 and 34.9 kg/m^2^, namely overweight type II or obesity type I [22]. To design a potentially successful program, basic behavior change aspects have been included: nutrition, physical activity, self-recording and goal setting [7, 23]. Perceived family and friends support were shown particularly helpful during the process of health behavior change [10, 11, 24], and it should be considered during treatment design. Finally, psychological aspects like dealing with negative self-talk must be driven by a healthcare professional, providing personalized feedback and a non-judgmental attitude [9–12]. One of the most tested lifestyle change intervention for weight management is the LEARN program [25]. The word LEARN is an acronym of the five main treatment areas: Lifestyle, Exercise, Attitudes, Relationships and Nutrition [25]. This program is a self-help book which includes basic treatment areas [7, 23], and also includes social support as a relevant aspect to achieve long-term lifestyle changes [10, 11, 24].

However, LEARN program presents two important limitations: i) it is administrated to one person at a time; ii) there is no healthcare professional to solve doubts and provide personalized feedback. Our web-based intervention is an online adaptation of LEARN program [25]. We respected the structure and contents of the original program. However, we adapt the nutrition recommendations to Mediterranean diet, more typical in Spain, and we update the delivery format to reach as many people as possible. In addition to the website, a mobile application was developed to record daily nutrition and exercise information, and weekly weight. To encourage participants a specialized psychologist provides weekly personalized feedback about website activity and mobile application records. This new approach of the LEARN program was named *en_línea.*

In Spain the estimated prevalence of overweight is 39.3 and 21.6% of general obesity in adults between 25 and 64 years old [26]. The main aim of *en_línea* is its application from Spanish primary care centers. Healthcare professionals of primary care centers are the first clinicians to assess and treat obesity [27]. High-intensity in-person interventions in primary care, both individual and group format, have shown effective weight loss results [28, 29]. Unfortunately, the large amount of obesity patients imposes a significant public health expense [30], making impossible to offer quality high-intensity in-person treatment to all affected population. Moreover, these treatment options present a limited reached, people who have to travel great distance to assist to the appointments are likely not to be interested or drop out at early stages [17, 20]. As there are a lot of people in wait lists for obesity treatments in public hospitals and primary care centers, *en_línea* could be a great option for these patients and for those who live away from the assistance centers.

A randomized controlled trial (RCT) is need to examine the effectiveness of *en_línea* comparing with a standard primary care group therapy and a control group. All conditions will be described later in detail. We hypothesize that *en_línea* group will obtain better or, at least, similar effectiveness results than the standard therapy group, and better results than the control group. The primary aim of the RCT will be determine the effectiveness weight loss and maintenance of *en_línea* with the two comparison groups. The secondary objectives to determine the effectiveness of *en_línea* in comparison with the other conditions will be: a) the adherence to intervention, i.e. website tasks and mobile application records; b) number and stage of drop outs. We will conduct a 17 weeks, parallel-group, randomized controlled trial. Randomization will be performed with a simple randomization with a 1:1:1 allocation ratio. The aims of this paper are to describe the development of *en_línea* program and the protocol for the RCT.

## Methods/design

**Fig. 1:**
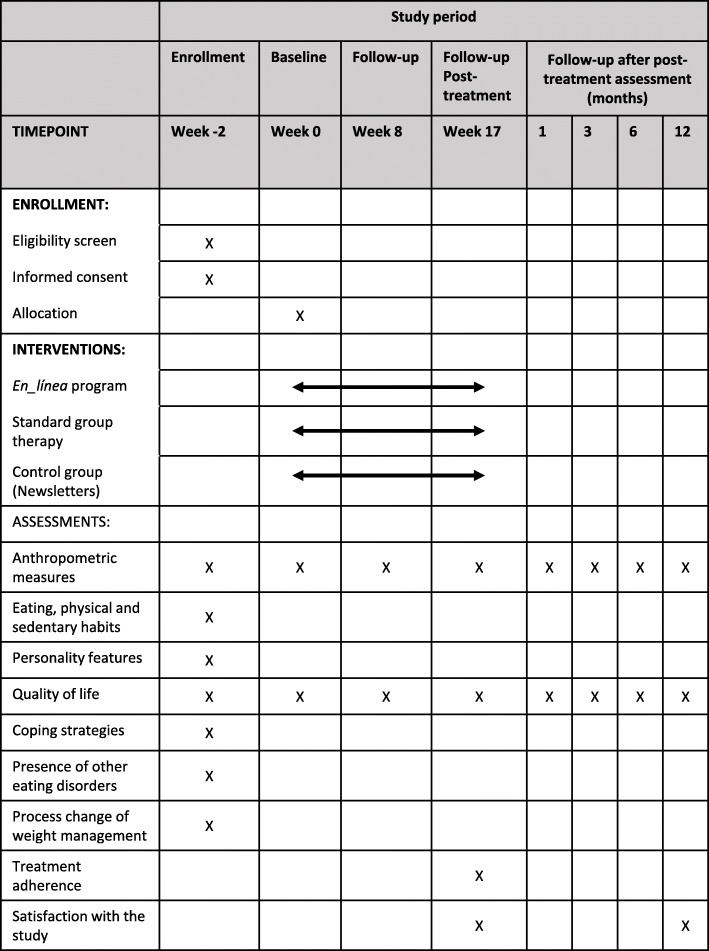
Overview of research schedule and procedure

This protocol is written according to SPIRIT 2013 guidance for protocols of clinical trials (Fig. 1) [31].

### Setting

The study will be conducted at the Unit of Behavior Therapy, a clinic service in the Faculty of Psychology of the University of Barcelona, located in Barcelona. The center is registered in the Sanitary Registration of the Health Department of the Government of Catalonia. The Unit of Behavior Therapy was opened in 1985 to provide psychological service to the community and to train new psychologists [32]. Currently, the center is staffed by psychologists in training, two supervisors and four experience psychologist. The clinic service director is a clinical psychologist expert in the study and treatment of eating disorders and obesity.

### Participants

The target population will be overweight type II or obese type I people, from the community and from the metropolitan area of Barcelona and surroundings. Participants are volunteers and they must sign the written informed consent before beginning with the investigation procedures.

#### Inclusion criteria

At randomization, eligible participants for the trial must comply all following requirements: 1) Overweight type II or obesity type I. BMI between 27 and 34.9 kg/m^2^; 2) Age between 18 and 65 years old; and, 3) Compliance with all evaluation phases.

#### Exclusion criteria

If eligible participants present at least one of the following criteria, they will be excluded from the trial: 1) Presence of several physical disease, i.e. diabetes, hypertension, cancer or metabolic disorders; 2) Presence of several psychological problem, i.e. depression, anxiety; 3) Presence of other eating disorders, i.e. anorexia nervosa, bulimia nervosa, binge eating disorders; 4) Use of drugs, i.e. slimming, anovulatory or psychotropic; 5) Pregnancy or planned pregnancy for the next 6 months; and, 6) Following another weight control program at the time of selection.

### Interventions

#### en_línea

It is a version of the LEARN program [25] delivered via online. It is not only a weight loss program, the main objective of *en_línea* is the acquirement of long-term healthy habits. The five treatment areas are: Lifestyle, Exercise, Attitudes, Relationships and Nutrition. We have designed and developed a website (www.programaenlinea.org) with 17 weekly treatment sessions and an exclusive mobile application for self-recording.

Participants will receive an email with two attached files to provide the instructions to use *en_línea* website and the mobile application. They will be supplied with a private username and password to access to their profiles. Weekly they will access to a new session. Session 0 is aimed to introduce the program, to get familiar with it and to self-assess the motivation level to involve in this process.

**Fig. 2:**
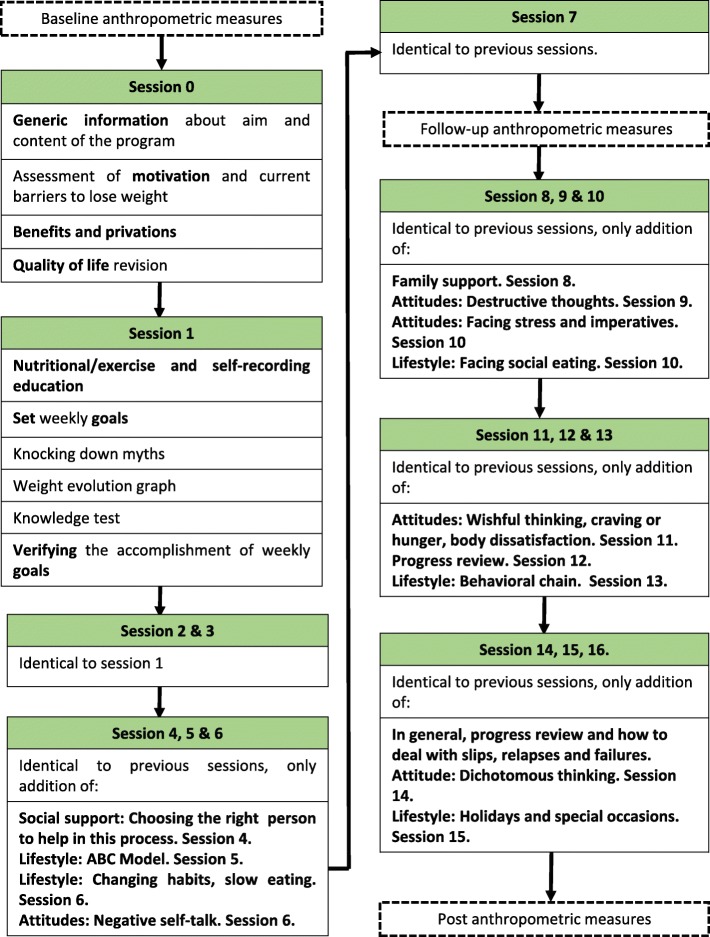
Overview of *en_línea* program content

The sessions from 1 to 17 present a similar structure, but the contents are getting harder. At the first three sessions, the information is basically nutrition, physical activity and self-recording. Session 4 adds a complete explanation about the importance of social support, and provide instructions to choose a correct confidence person to help during the program. Lifestyle and attitudes contents will developed through the next sessions, when the basic components are already consolidated. Figure 2 shows an overview of the content of intervention. In the figure, the sentence “identical to previous sessions” means that the structure of each session is the same as in session 1. Nutrition and exercise are treated each week, including new aspects and consolidating the old ones.

“Set weekly goals” and “Verifying weekly goals” sections are aimed to adjust expectations and establish realistic goals. If the participants, at the end of the week, verify the accomplishment of 75% of the initial weekly goals, they will receive an automatic congratulation message and they should reward themselves.

Participants will download a mobile application designed exclusively for *en_línea*. They should record their daily meals, at least five records per day; their daily physical activity, at least their daily steps; and, their weekly weight. To count their daily steps participants will use commercial mobile applications.

Finally, once a week a specialized psychologist will provide personalized feedback about the activities of the website and the mobile application records. There is a space for this feedback at the end of each section of the website. The participant can review each activity with the corresponding feedback. Participants can send an email to the psychologist to solve doubts.

#### Standard group therapy

Participants in this arm will received a 10 sessions of standard primary care group therapy. Sessions 1 and 2 will be weekly and sessions from 3 to 10 will be biweekly. Sessions, between 8 and 10 participants, will be conducted by a specialized psychologist and they will last 90 min. Treatment areas will be the same as *en*_*línea,* beginning with nutrition and exercise and a progressive incorporation of the other topics. Material to work at home and self-recording will be provided in paper. The structure of the sessions will be: home activities and self-recording review, new information and strategies, and establishment of activities for the week. Anthropometric measures will be taken at the same time as *en_línea.*

#### Control group

Participants in the control group only will receive a biweekly newsletter by email without feedback. They will be provided with material and instructions to self-record their daily meals and exercise. The newsletter only will content basic information about nutrition and exercise.

Follow-up sessions will take place in three arms to assess the adherence to the interventions and to evaluate the main objective of this study, the acquirement of long-term healthy habits. These face-to-face sessions will last between 15 and 30 min and will take place 1, 3, 6 and 12 months after the post treatment session. In this follow-ups, we will review: anthropometric measures, quality of life, nutrition and exercise habits.

### Outcomes

#### Primary outcomes measures

The primary outcome is the weight loss. First, differences between the pre and post measures for each treatment arm. Treatment success is defined as 5–10% of weight loss respect the initial weight. Then, significant differences in weight loss and other anthropometric measures, specified later, between the three treatment arms. Finally, the maintenance of weight loss during the follow-ups, significant differences between the three treatment arms.

#### Secondary outcomes measures

To determine the effectiveness of the different interventions following results will be considered for each intervention arm: 1) Adherence: at least 80% of completed website activities and 80% of self-records; 2) Drop out: Participants must finish the treatment; 3) Quality of life: significant differences between pre-post and follow-ups measures. Moreover, significant differences between the three interventions for the three variables aforementioned.

At the end of the study, participants will receive an email with a satisfaction questionnaire with the website and the mobile application, with an available space to make improvement suggestions.

### Study design and randomization

#### Procedure

Participants will be recruited through advertising (flyers, posters, University website, workplace-based emails and social networks). Obese and overweight people interested in participating in the study must send an email to the coordinator of the project. Participants will receive an email to attend an appointment with a psychologist. In this first appointment, patients will be informed about the aims and characteristics of the study, they must sign the written informed consent and, after that, a brief interview and baseline anthropometric measures will be taken. Anthropometric measures will be weight, height; and, neck, chest, waist, hips, leg and arm circumferences. All measures will be taken with the participant wearing light clothing and without shoes. Height will be taken with 1 cm using a stadiometer. Weight will be measured on a digital scale. Circumferences measurements will be measure following the indications provided by WHO [33].

To ensure the accomplishment of inclusion and exclusion criteria participants must answer a large battery of questionnaires specified later. These questionnaires are divided in two batches. After the first in-person appointment, participants will receive an email with the instructions to complete the first batch of questionnaires, they will be provided with an identity number for data protection and with the direct link to the available questionnaires in the platform SurveyMonkey. After the completion of the first part, a second email with the link to the second batch of questionnaires will be sent.

**Fig. 3:**
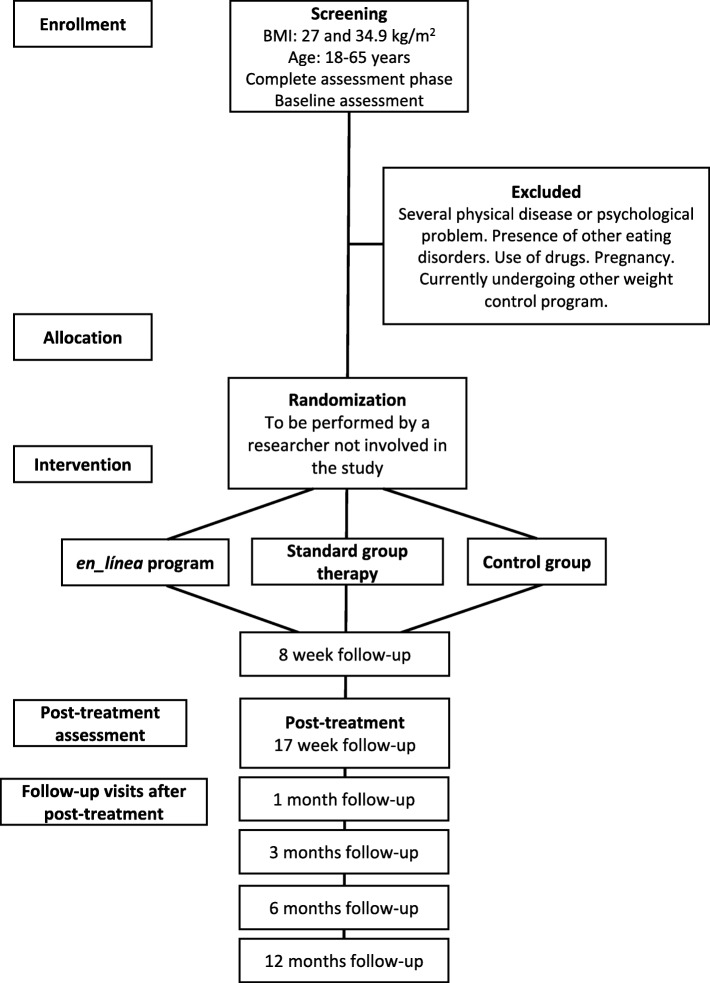
Flow diagram of *en_línea* study

Assessment phase will last 2 weeks, selected participants will be randomized to one of the three study arms. Patients will receive an email with information about their belonging group. Obese and overweight people randomized to *“en_línea”* program, must assist to a 1 h training session to get familiar with the website and the mobile application. When participants are included in the study, both *en_línea* and group intervention, they will be given timings for follow-up meetings during the treatment at week 8 and week 17. After interventions finish, follow-up meetings will be happened at 1, 3, 6 and 12 months. In the follow-up visits a brief interview and anthropometric measures will be taken again, the duration of these meeting will be between 15 and 30 min (Fig. 3).

The first evaluation and follow-up visits will be conducted by the coordinator of the project and the interventions will be conducted by two psychologists. The psychologists involved are all certified general sanitary psychologist training for the interventions chosen for this investigation.

#### Randomization and blinding

Simple randomization will be applied using computer-generated random numbers [34], with a 1:1:1 allocation ratio. Simple randomization has proven to be reliable to large clinical research, generating similar numbers of subjects per group [34]. Participants will be randomly assigned to *en_línea* program, standard group therapy or control group. Randomization will be carried out by an independent researcher of the study. Therapists will not know the patient allocation until the coordinator inform them to begin the treatment. Thus, blinding will not be possible for the therapists during the intervention.

Currently, this study is in the recruitment phase. No statistical analysis have been performed at this time.

#### Assessment instruments

The questionnaires used in the assessment phase, apart from a sociodemographic questionnaire ad hoc*,* will be the following in its Spanish version:

- E-TONA structured interview: a self-reported adaptation for adults will be used. This tool was designed to assess behavioral eating habits, physical activity, sedentary behaviors and health problems in fathers and children [35]. Only the behavioral eating habits data will be used in this study. The items of this descriptive and no diagnostic interview are multiple or dichotomous choice.
- The Temperament and Character Inventory Revised (TCI-R) [36, 37]. 240 items answered in a 5-Likert scale (1 = completely false; 5 = completely true). It includes seven main dimensions, 4 temperaments and 3 characters, with their corresponding specific features.
- Bulimic Investigatory Test Edinburgh (BITE) [38, 39]. Self-reported 33 item questionnaire, designed to identify bulimic symptoms: presence of binge eating and symptoms severity. Items are answered on different Likert scales or dichotomous choice.
- Stages of Change for Weight Management (S-Weight) [40]. Questionnaire of 5 excluding items to classify participants in one of the 5 stages of change for weight management.
- Process of Change for Weight Management (P-Weight) [40]. 33 items answered in a 5-point Likert scale to assess attitudes and behaviors to control weight.
- Night Eating Questionnaire (NEQ) [41, 42]. Self-reported 14 items scale to assess behavioral and psychological symptoms of Night Eating Disorder.
- SF-36 Health Survey (SF-36) [43, 44]. 36 items to assess functional health and wellbeing from the perspective of the respondent. It includes 8 scales related with the main aspects of heath, both physical and mental health.
- Coping Inventory Strategies (CSI) [45, 46]. 40 items tool answered on a 5-Likert scale (0 = not at all; 4 = totally), to assess 8 different coping strategies.
- Dutch Eating Behavior Questionnaire (DEBQ) [47, 48]. 33 item questionnaire answered in a 5-point Likert scale (1 = never; 5 = very often), to assess different types of intake.
- Depression Anxiety Stress Scale (DASS-21) [49, 50]. 21 item questionnaire answered in a 5-point Likert scale (0 = nothing applicable to me; 5 = applicable to me most of the time), to evaluate the severity of depression, anxiety and stress experimented during the last week.

### Statistical analysis

To estimate the sample size has been used the primary outcome, weight loss differences between baseline and post-treatment measures. The range of the effect size of the intervention will be 0.5 SD in group comparisons, alpha 5% and two-tail tests [51]. These data determine that we need at least 88 patients per group, considering an expected 31% dropout rate [52], our final choice will be *N* = 305 [53].

Analysis will be performed using STATA/IC 14.2. All data will be checked for missing values, accuracy and normality criteria. Intention to treat analysis (ITT) will be used to analyze the data, including all randomized participants. After that, per protocol analysis will be performed for participants who have completed 17 weeks follow-up. ANOVA for repeated measures, with type of intervention as independent variable and time (baseline and follow-up visits) as dependent variable, will be conducted to analyze primary and secondary outcomes.

## Discussion

The high obesity rates [1, 2] are an evidence that current interventions are not effective enough and new approaches are necessary [7–11]. Internet and new technologies have become essential tools in our daily routine [15]. An overuse of new technologies could trigger unhealthy behaviors [12–14] or an addition to these new resources. However, properly used Internet could be a powerful way to engage the patients in innovative treatments [16].

The future challenge is to get over the limitations of traditional weight control programs: poor long-term results and adherence rates [7–9]. Moreover, to design a new intervention the focus have to change from the disease to understand the people who is suffering the problem and their environment [10]. Thus, social support and personalized feedback should be relevant aspects to consider designing new obesity treatments [10–12]. High-intensity in-person treatments have accomplished the best results in primary care, but the large amount of people suffering obesity makes impossible offer this kind of interventions to all affected population [27–30].

*en_línea* is a project designed to be an alternative in primary care. The target population is people who are waiting for a treatment or who have to cover large distance to keep an appointment. This new approach try to consider all the aforementioned limitations. The intervention is based on the LEARN program [25], a tested weight control program, which includes social support as a relevant treatment area [10–12, 24]. *en_línea* is an online version with its own website and mobile application to reach as many obese people as possible at the same time. Online therapies are not only useful for that people who live far away [17–20]. This new intervention format is appropriate for patients who prefer not to share their intimate problems with a group, and for the new generations who feel comfortable using new technologies. Thus, a program focused on the person and their environment [10–14], with social support as a treatment area [10, 11], delivered by healthcare professionals providing personalized feedback [9–11], and with an online format [21], should provide better long-term and adherence results.

Finally, *en_línea* is the results of taking advantage of the daily use of new technologies and including them as health tools. Moreover, we reviewed previously successful weight control programs and we adapt their best features, trying to cover their limitations at the same time. Improving current treatment is the future. However, we must not forget that changing habits and losing weight are very difficult processes. Therefore, an evolution to a nonjudgmental and supportive society is necessary.

## Data Availability

Data sharing is not applicable to this article because as no datasets were generated or analyzed during the current study.

## Abbreviations

BITE: Bulimic Investigatory Test Edinburgh
BMI: Body Mass Index
CSI: Coping Inventory Strategies
DASS: Depression Anxiety Stress Scale
DEBQ: Dutch Eating Behavior Questionnaire
LEARN: Lifestyle, Exercise, Attitudes, Relationships and Nutrition.
NEQ: Night Eating Questionnaire
P-Weight: Process of Change for Weight Management
RCT: Randomized Controlled Trial
SF-36: SF-36 Health Survey
S-Weight: Stages of Change for Weight Management
TCI-R: Temperament and Character Inventory Revised
WHO: World Health Organization

## References

[1] NCD Risk Factor Collaboration. Trends in adult body-mass index in 200 countries from 1975 to 2014: a pooled analysis of 1698 population-based measurement studies with 19.2 million participants. Lancet. 2016; 10.1016/S0140-6736(16)30054-X. 10.1016/S0140-6736(16)30054-X PMC7615134 27115820

[2] World Health Organization: Obesity and overweight. https://www.who.int/en/news-room/fact-sheets/detail/obesity-and-overweight. Accessed 25 Mar 2019.

[3] James PT. Obesity: the worldwide epidemic. Clin Dermatol. 2004. 10.1016/j.clindermatol.2004.01.010. 10.1016/j.clindermatol.2004.01.010 15475226

[4] Rajan TM, Menon V. Psychiatric disorders and obesity: a review of association studies. J Postgrad Med. 2017. 10.4103/jpgm.JPGM_712_16. 10.4103/jpgm.JPGM_712_16 PMC5525483 28695871

[5] Puhl RM, Brownell KD. Confronting and coping weight stigma: an investigation of overweight and obese adults. Obesity. 2006. 10.1038/oby.2006.208. 10.1038/oby.2006.208 17062811

[6] O’Brien KS, Latner JD, Puhl RM, Vartanian LR, Giles C, Griva K, Carter A. The relationship between weight stigma and eating behaviors is explained by weight bias internalization and psychological distress. Appetite. 102:70–6. 10.1016/j.appet.2016.02.032. 10.1016/j.appet.2016.02.032 26898319

[7] Coons MJ, DeMott A, Buscemi J, Duncan JM, Pellegrini CA, Steglitz J, Pictor A, Spring B (2012). Technology interventions to curb obesity: a systematic review of the current literature. Curr Cardiovasc Risk Rep.

[8] Beleigoli AM, Andradade AQ, Cançado AG, Paulo MN, Diniz MF, Ribeiro AL (2019). Web-based digital health interventions for weight loss and lifestyle habit changes in overweight and obese adults: systematic review and meta-analysis. J Med Internet Res.

[9] Sherrington A, Newham JJ, Bell R, Adamson A, McColl E, Araujo-Soares V. Systematic review and meta-analysis of internet-delivered interventions providing personalized feedback for weight loss in overweight and obese adults. Obes Rev. 2016. 10.1111/obr.12396. 10.1111/obr.12396 PMC4999041 26948257

[10] Rand K, Vallis M, Aston M, Price S, Piccinini-Vallis H, Rehman L, Kirk SF. It is not the diet; it is the mental part we need help with. A multilevel analysis of psychological, emotional, and social well-being in obesity. Int J Qual Stud Health Well-bein. 2017. 10.1080/17482631.2017.1306421. 10.1080/17482631.2017.1306421 PMC5421368 28418818

[11] Kirk SF, Price SL, Penney TL, Rehman L, Lyons RF, Piccinini-Vallis H, Vallis TM, Curran J, Aston M. Blame, shame and lack of support: a multilevel study on obesity management. Qual Health Res. 2014. 10.1177/1049732314529667. 10.1177/1049732314529667 24728109

[12] Smolak L, Chun-Kennedy C, Choate LH (2013). Sociocultural influences on the development of eating disorders and obesity. Eating disorders and obesity: A counserlor’s guide to prevention and treatment.

[13] Vandelanotte C, Sugiyama T, Gardiner P, Owen N. Associations of leisure-time and computer use with overweight and obesity, physical activity and sedentary behaviors: cross-sectional study. J Med Internet Res. 2009. 10.2196/jmir.1084. 10.2196/jmir.1084 PMC2762849 19666455

[14] Sutherland LA, Wildemuth B, Campbell MK, Haines PS. Unraveling the web: an evaluation of the content quality, usability, and readability of nutrition web sites. J Nutr Educ Behav. 2005. 10.1016/S1499-4046(06)60160-7. 10.1016/s1499-4046(06)60160-7 16242061

[15] Internet World Stats: World internet users and 2019 population stats. https://www.internetworldstats.com/stats.htm. Accessed 28 Mar 2019.

[16] Spruijt-Metz D, Wen CK, O’Reilly G, Li M, Lee S, Emken BA, Mitra U, Annavaram M, Ragusa G, Narayanan S. Innovations in the use of interactive technology to support weight management. Curr Obes Rep. 2015. 10.1007/s13679-015-0183-6. 10.1007/s13679-015-0183-6 PMC4699429 26364308

[17] Walthouwer MJ, Oenema A, Soetens K, Lechner L, De Vries H. Systematic development of a text-driven and a video-driven computer-tailored obesity prevention intervention. BMC Public Health. 2013. 10.1186/1471-2458-13-978. 10.1186/1471-2458-13-978 PMC4015713 24138937

[18] Hutchesson MJ, Rollo ME, Krukowski R, Ells L, Morgan PJ, Callister R, Plotnikoff R, Collins CE. eHealth interventions for the prevention and treatment of overweight and obesity in adults: a systematic review with meta-analysis. Obes Rev. 2015. 10.1111/obr.12268. 10.1111/obr.12268 25753009

[19] Oosterveen E, Tzelepis F, Ashton L, Hutchesson J (2017). A systematic review of eHealth behavioral interventions targeting smoking, nutrition, alcohol, physical activity and/or obesity for young adults. Prev Med.

[20] Afshin A, Babalola D, Mclean M, Yu Z, Ma W, Chen C, Arabi M, Mozaffarian D. Information technology and lifestyle: a systematic evaluation of internet and mobile interventions for improving diet, physical activity, obesity, tobacco and alcohol use. J Am Heart Assoc. 2016. 10.1161/JAHA.115.003058. 10.1161/JAHA.115.003058 PMC5079005 27581172

[21] Matthews L, Pugmire J, Moore L, Kelson M, McConnachie A, McIntosh E, Morgan-Trimmer S, Murphy S, Hughes K, Coulman E, Utkina-Macaskill O, Simpson SA. Study protocol for the “HelpMeDoIt!” randomized controlled feasibility trial: an app, web and social support-based weight loss intervention for adults with obesity. BMJ Open. 2017. 10.1136/bmjopen-2017-017159. 10.1136/bmjopen-2017-017159 PMC5665248 29074513

[22] World Health Organization: Body mass Index. http://www.euro.who.int/en/health-topics/disease-prevention/nutrition/a-healthy-lifestyle/body-mass-index-bmi. Accessed 02 Apr 2019.

[23] Michie S, Abraham C, Whittington C, McAteer J, Gupta S. Effective techniques in healthy eating and physical activity interventions: a meta-regression. Health Psychol. 2009. 10.1038/a0016136. 10.1037/a0016136 19916637

[24] Karfopoulou E, Anastasiou CA, Avgeraki E, Kosmidis MH, Yannakolia M. The role of social support in weight loss maintenance: results from the MedWeight study. J Behav Med. 2016. 10.1007/s10865-016-9717-y. 10.1007/s10865-016-9717-y 26801339

[25] Brownell KD (2004). The LEARN program for weight management.

[26] Aranceta-Bartrina J, Pérez-Rodrigo C, Alberdi-Aresti G, Ramos-Carrera N, Lázaro-Masedo S. Prevalence of general obesity and abdominal obesity in the Spanish adult population (aged 25-64 year) 2014-2015: the ENPE study. Rev Esp Cardiol. 2016. 10.1016/j.rec.2016.02.009. 10.1016/j.rec.2016.02.009 27133458

[27] Tsai A, Remmert J, Butryn ML, Wadden TA. Treatment of obesity in primary care. Med Clin N Am. 2017. 10.1016/j.mcna.2017.08.005. 10.1016/j.mcna.2017.08.005 29156186

[28] Wadden TA, Butryn ML, Hong PS, Tsai A. Behavioral treatment of obesity in patients encountered in primary care settings: a systematic review. JAMA. 2014. 10.1001/jama.2014.14173. 10.1001/jama.2014.14173 PMC4443898 25369490

[29] Leblanc ES, O’Connor E, Whitlock EP, Patnode CD, Kapka T. Effectiveness of primary care-relevant treatments for obesity in adults: a systematic evidence review for the U.S. preventive services task force. Ann Intern Med. 2011. 10.7326/0003-4819-155-7-201110040-00006. 10.7326/0003-4819-155-7-201110040-00006 21969342

[30] Tremmel M, Gerdtham U, Nilsson P, Saha S. Econominc burden of obesity: a systematic literature review. Int J Environ Res Public Health. 2017. 10.3390/ijerph14040435. 10.3390/ijerph14040435 PMC5409636 28422077

[31] Chan A, Tetzlaff JM, Gøtzsche PC, Altman DG, Mann H, Berlin JA, Dickersin K, Hróbjartsson A, Schulz KF, Parulekar WR, Krleza-Jeric K, Laupacis A, Moher D (2013). SPIRIT 2013 explanation and elaboration: guidance for protocols of clinical trials. BMJ.

[32] Bados A, Balaguer G, Saldaña C. Outcome of cognitive-behavioural therapy in training practice with anxiety disorder patients. Brit J Clin Psychol. 2007. 10.1348/014466507X209961. 10.1348/014466507X209961 17535537

[33] World Health Organization (2017). The WHO STEPwise approach to Surveillance of noncommunicable diseases (STEPS).

[34] Suresh KP. An overview of randomization techniques: an unbiased assessment of outcome in clinical research. J Hum Reprod Sci. 2011. 10.4103/0974-1208.82352. 10.4103/0974-1208.82352 PMC3136079 21772732

[35] Saldaña C (2010). Entrevista para la evaluación de comportamiento alimentario y actividad física en niños y adolescentes, version padres. Proyecto E-TONA.

[36] Cloninger C. The Temperament and Character Inventory-Revised. St. Louis, MO: Center for Psychobiology of Personality, Washington University; 1999.

[37] Gutierrez-Zotes JA, Bayón C, Montserrat G, Valero J, Labad A, Cloninger C, Fernández-Aranda F (2004). Inventario de Temperamento y el Carácter-Revisado (TCI-R). Baremación y datos normativos en una muestra de población general. Actas Esp Psiquiatr.

[38] Henderson M, Freeman CP (1987). Self-rating scale for bulimia: the BITE. Br J Psychol.

[39] Vaz FJ, Peñas EM (1999). Differential study of the complete and subclinical presentations of bulimia nervosa. Actas Esp Psiquiatr.

[40] Andrés A, Saldaña C, Gómez-Benito J. The transtheoretical model in weight management: validation of the processes of change questionnaire. Obes Facts. 2011. 10.1159/000335135. 10.1159/000335135 PMC6444774 22248993

[41] Allison KC, Lundgren JD, O’Reardon JP, Martino NS, Sarwer DB, Wadden TA, Crosby RD, Engel SG, Stunkard AJ. The Night Eating Questionnaire (NEQ): psychometric properties of a measure of severity of the night eating syndrome. Eat Behav. 2008. 10.1016/j.eatbeh.2007.03.007. 10.1016/j.eatbeh.2007.03.007 18167324

[42] Moizé V, Gluck ME, Torres F, Andreu A, Vidal J, Allison K (2012). Transcultural adaptation of the Night Eating Questionnaire (NEQ) for its use in the Spanish population. Eat Behav.

[43] Ware JE, Snow KK, Kosinski M, Gandek B. SF-36 Health Survey: manual and interpretation guide. Boston: Nimrod Press; 1993.

[44] Alonso J, Prieto L, Antó JM (1995). La versión española del SF-36 Health Survey (Cuestionario de salud SF-36): un instrumento para la medida de los resultados clínicos. Med Clin (Barc).

[45] Tobin D, Holroyd K, Reynols R, Kigal J. The hierarchical factor structure of Coping Strategies Inventory. Cognitive Ther Res. 1989. 10.1007/BF01173478.

[46] Cano F, Rodríguez L, García J (2007). Adaptación española del Inventario de Estrategias de Afrontamiento. Actas Esp Psiquiatr.

[47] Van Strien T, Frijters J, Bergers G, Defares P. The Dutch Eating Behavior Questionnaire (DEBQ) for assessment of restrained, emotional and external eating behaviors. Eat Behav. 1986. 10.1002/1098-108X(198602)5:2<295::AID-EAT2260050209>3.0.CO;2-T.

[48] Cebolla A, Barrada JR, Van Strien T, Oliver E, Baños R. Validation of the Dutch Eating Behavior Questionnaire (DEBQ) in a sample of Spanish women. Appetite. 2014. 10.1016/j.appet.2013.10.014. 10.1016/j.appet.2013.10.014 24177441

[49] Lovibond SH, Lovibond PF. Manual for the Depression Anxiety Stress Scales (DASS). New South Wales: Psychology Foundation Monograph; 1993.

[50] Bados A, Solanas A, Andrés R. Psychometric properties of the Spanish version of depression, anxiety and stress scales (DASS). Psicothema. 2005;17:679–83.

[51] Gogtay NJ. Principles of sample size calculation. Indian J Ophthalmol. 2010. 10.4103/0301-4738.71692. 10.4103/0301-4738.71692 PMC2993982 20952836

[52] Melville KM, Casey LM, Kanavagh DJ. Dropbout from internet-based treatment for psychological disorders. Br J Clin Psychol. 2010. 10.1348/014466509X472138. 10.1348/014466509X472138 19799804

[53] G*Power: Statistical Power Analysis for Windows and Mac. http://www.gpower.hhu.de/. Accessed 10 Apr 2019.

